# Current provision of general practitioner services in or alongside emergency departments in England

**DOI:** 10.1136/emermed-2020-210539

**Published:** 2021-02-22

**Authors:** Heather Brant, Sarah Voss, Katherine Morton, Alison Cooper, Michelle Edwards, Delyth Price, James Gaughan, Adrian Edwards, Jonathan Benger

**Affiliations:** 1 Faculty of Health and Applied Sciences, University of the West of England, Bristol, UK; 2 Division of Population Medicine, School of Medicine, Cardiff University, Cardiff, UK; 3 Centre for Health Economics, University of York, York, North Yorkshire, UK; 4 Division of Population Medicine, School of Medicine, Cardiff University, Cardiff, South Glamorgan, UK

**Keywords:** emergency care systems, primary care, urgent care, emergency department

## Abstract

**Background:**

In 2017, general practitioners in or alongside the emergency department (GPED), an approach that employs GPs in or alongside the ED to address increasing ED demand, was advocated by the National Health Service in England and supported by capital funding. However, little is known about the models of GPED that have been implemented.

**Methods:**

Data were collected at two time points: September 2017 and December 2019, on the GPED model in use (if any) at 163/177 (92%) type 1 EDs in England. Models were categorised according to a taxonomy as ‘inside/integrated’, ‘inside/parallel’, ‘outside/onsite’ or ‘outside/offsite’. Multiple data sources used included: on-line surveys, interviews, case study data and publicly available information.

**Results:**

An increase of EDs using GPED was observed from 81% to 95% over the study period. ‘Inside/parallel’ was the most frequently used model: 30% (44/149) in 2017, rising to 49% (78/159) in 2019. The adoption of ‘inside/integrated’ models fell from 26% (38/149) to 9% (15/159). Capital funding was received by 87% (142/163) of the EDs sampled. We identified no significant difference between the GPED model adopted and observable characteristics of EDs of annual attendance, 4-hour wait, rurality and deprivation within the population served.

**Conclusion:**

The majority of EDs in England have now adopted GPED. The availability of capital funding to finance structural changes so that separate GP services can be provided may explain the rise in parallel models and the decrease in integrated models. Further research is required to understand the relative effectiveness of the various models of GPED identified.

**Key messages**

**What is already known on this subject**

- Emergency departments (EDs) in the UK have faced unprecedented demand with waiting times at record levels.
- It has been estimated that between 15% and 40% of patients attending the ED could be managed by general practitioners (GPs).
- In 2017, National Health Service policy advocated the introduction of GPs in or alongside the ED (GPED), supported by the provision of capital funding,

**What this study adds**

- At the time the policy was advocated most EDs already had a model of GPED in place.
- Using multiple data sources to determine the model of GPED model in 177 type I EDs, we found that between September 2017 and December 2019 the number of EDs with a GPED service increased; parallel GPED services became more common, while the number of integrated services fell.
- We found no association between the type of GPED model adopted and the observable characteristics of EDs in England.

## Introduction

During 2019, attendances to emergency departments (EDs) in the National Health Service (NHS) reached record levels. The year 2018–2019 saw an increase of 4.4% compared with 2017–2018, and 21% since 2009–2010.^1^ It has been estimated that between 15% and 40% of patients attending the ED could be managed by the general practitioners (GPs).^2 3^ In 2015, The ‘Keogh Review’ of urgent care recommended colocating GPs alongside EDs to filter patients with primary care problems to alternative providers,^4^ despite a lack of supporting research evidence.^5^ At that time, a proportion of EDs across England had already implemented a range of new models of care with some form of GP colocation reported in 43%.^2^

**Figure 1:**
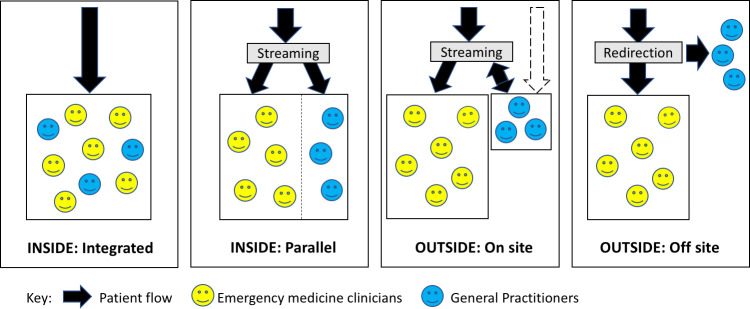
Taxonomy of general practice service models in or alongside the emergency department. Adapted from Cooper *et al* 8.

In March 2017, £100 million of capital funding was allocated in the UK Chancellor’s budget to support the introduction of GPs in or alongside the ED (GPED) by October 2017.^6 7^ However, little is known about the effect of this initiative on the actual provision of GPED services. This paper describes the provision of GPED models at the time of policy change (September 2017, prior to the intended implementation deadline of October 2017) and 2 years later (December 2019) in England. Models were classified according to an iteratively developed taxonomy (figure 1).^8^

## Methods

Data were collected on the GPED model(s) provided by all 177 type 1 EDs (consultant-led 24-hour services with full resuscitation facilities) in England. Sources included an online survey conducted by Cardiff University,^9^ and a combined interview study and online survey conducted by the University of the West of England (UWE).^10^ These data included interviews with clinical leads from the EDs that had applied for capital funding and information collected during the UWE GPED study from case study sites. This was supplemented by data sourced from public websites and NHS England.

Data were collected at two time points: September 2017 and December 2019, and collated in a single database. Models were classified into one of four types according to an iteratively developed taxonomy: inside/integrated, inside/parallel, outside/onsite, outside/offsite (figure 1).^8^

We conducted pairwise comparisons of the characteristics of the EDs (number of attendances, proportion treated within 4 hours, deprivation, rurality and capital funding) for each of the models of GP collaboration. Two-sided t-tests were used to compare means with a significance level of 0.05.

### Patient and public involvement

A patient and public contributor group was involved in study design, project management and dissemination. Members of this group joined the study steering committee, assisted in the preparation of patient-facing and other study materials and attended a series of workshops to contribute to data interpretation and comment on emerging research findings.

## Results

Data were obtained from 163/177 (92%) of all type 1 EDs in England:

- 149/177 (84%) at September 2017.
- 159/177 (90%) at December 2019.
- 139/177 (79%) at both time points.

The GPED models in place in September 2017 and December 2019 are shown in table 1. Capital funding was awarded to 87% (142/163) of the participating EDs.

**Table 1:** General practitioner service models in or alongside the emergency department at the two time points studied

Model	September 2017 (n=149)	December 2019 (n=159)
Inside/integrated	38/149 (26%)	15/159 (9%)
Inside/parallel	44/149 (30%)	78/159 (49%)
Outside/onsite	33/149 (22%)	55/159 (35%)
Outside/offsite	5/149 (3%)	2/159 (1.3%)
No GP streaming	28/149 (19%)	8/159 (5%)
Use of two models	Parallel and offsite=1/149 (0.7%)	Integrated and on-site=1/159 (0.6%)
		Integrated and parallel=1/159 (0.6%)

GP, general practitioner.

Between September 2017 and 2019, 23 sites commenced and four sites ceased GPED provision. Three of those who ceased chose to discontinue an Inside/integrated model. The most common service change (20 sites) was from an inside/integrated to an inside/parallel model. Additionally, 11 sites moved from an outside/onsite to an inside/parallel model.

Table 2 shows the differences between group means of observed characteristics by GPED model choice and between each GPD model and no model. The p values from two-way t-tests of differences between group means are also presented. We found no significant (p<0.05) difference between group means by the type of GPED model adopted and the observable characteristics of included EDs (annual number of new attendances, proportion of patients treated within 4 hours deprivation and rurality of the population served and receipt of capital funding). Comparisons with off-site models were not made, due to the small number of observations in this group.

**Table 2:** Pairwise comparisons of observed characteristics by chosen GPs in or alongside the emergency department model and two-sided t-tests

September 2017										
	No of attendances		Proportion of patients treated within 4 hours		Deprivation within the local population		Rurality of the locality		Capital	
	Dif	P value	Dif	P value	Dif	P value	Dif	P value	Dif	P value
Integrated versus parallel	−11130	0.360	0.015	0.234	−1.994	0.284	0.048	0.126		
Integrated versus on site	−15330	0.308	0.001	0.937	−3.266	0.071	0.058	0.107		
Integrated versus no GP	19 330	0.073	0.015	0.251	1.255	0.479	−0.037	0.338		
Parallel versus on site	−4200	0.773	−0.014	0.337	−1.273	0.477	0.010	0.747		
Parallel versus no GP	30 460	0.003	0.000	0.997	3.249	0.070	−0.085	0.015		
On site versus no GP	34 660	0.012	0.014	0.350	4.521	0.010	−0.094	0.015		
December 2019										
Integrated versus parallel	−17005	0.162	−0.011	0.576	0.870	0.661	0.004	0.927	0.049	0.528
Integrated versus On site	−26161	0.054	−0.016	0.415	2.549	0.214	0.014	0.775	0.081	0.332
Integrated versus No GP	25 085	0.111	−0.032	0.148	5.483	0.019	−0.068	0.373	0.058	0.688
Parallel versus on site	−9156	0.395	−0.005	0.615	1.679	0.221	0.010	0.692	0.033	0.592
Parallel versus no GP	42 090	0.007	−0.022	0.160	4.614	0.011	−0.072	0.269	0.010	0.943
On site versus no GP	51 246	0.002	−0.017	0.287	2.934	0.094	−0.082	0.218	−0.023	0.867

GP, general practitioner.

## Discussion

Our findings indicate that the vast majority of EDs in England now include a colocated general practice service, most commonly parallel with ED provision. Fully integrated models tended to be replaced by a more complex and distinct general practice service component, possibly as a result of capital funding allocations that allowed structurally separate facilities to be established and attracted the involvement of community care providers. However, we found no significant differences between the GPED model adopted and the observable characteristics of an ED.

Previous research reported that 43% of EDs had a GP service in 2015,^2^ therefore, the increase in adoption in the 2 years before our study (from 43% to 81% of EDs) exceeded the increase in the 2-year period following the capital funding allocation (from 81% to 95%). Nevertheless, after the NHS policy announcement and associated capital funding, GPED became almost universally established.

### Limitations

This is the most complete and detailed mapping of GPED provision across England that has been published to date. However, the reliability of the data sources varied and required some interpretation by the research team. Further, data collection relied on self-report and the ability of respondents to accurately categorise their service provision into the taxonomy.

## Conclusion

The vast majority of EDs in England now have a GPED model in place. Central direction supported by capital funding may have resulted in an increase in parallel GPED models and a corresponding reduction in integrated approaches. Although it was possible to determine information about the use of GPED across time, the findings do not indicate why these models were chosen, and our analysis found no relationship between the type of model and the receipt of capital funding or other observable characteristics of the ED. Further research is required to understand the reasons for change and the relative clinical and cost effectiveness of different approaches to GPED provision.^5^

## Data Availability

Data are available on reasonable request. All data requests should be submitted to the corresponding author for consideration. Access to anonymised data may be granted following review.
